# Supplementary figures and images for: Ancient divergence time estimates in Eutropis rugifera support the existence of Pleistocene barriers on the exposed Sunda Shelf
Source: PeerJ. 2017 Oct 27;5:e3762. doi: 10.7717/peerj.3762 (PMC5661453; doi:10.7717/peerj.3762)

Bayesian Posterior Probability/ML Bootstrap

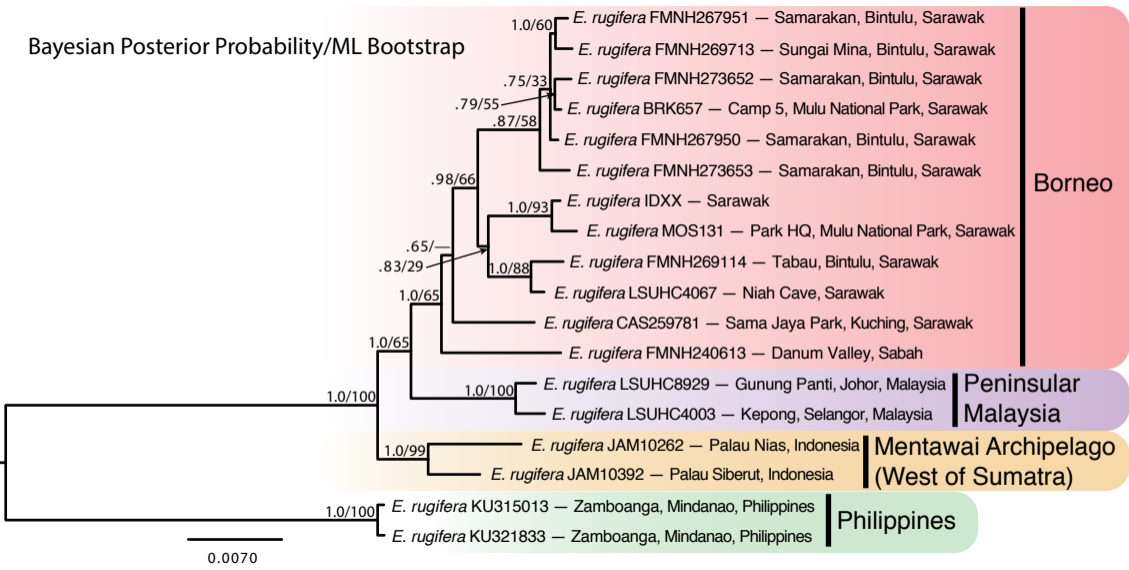

Supplement: Figure S1 — Support for the ML phylogeny indicated by bootstrap values (proportion of 1,000) and for the BI tree by posterior probabilities. [file peerj-05-3762-s001.pdf]

ND2

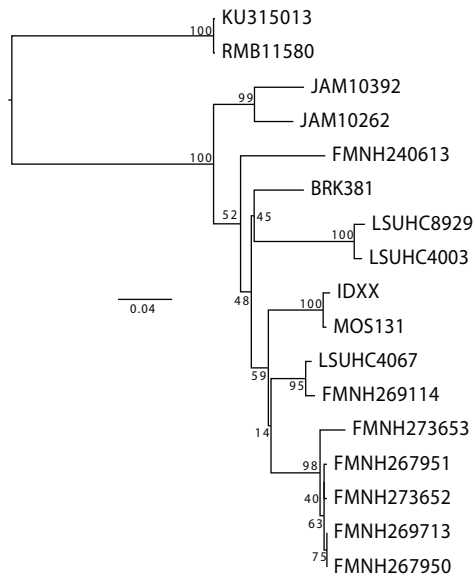

Nuclear - Concatenated

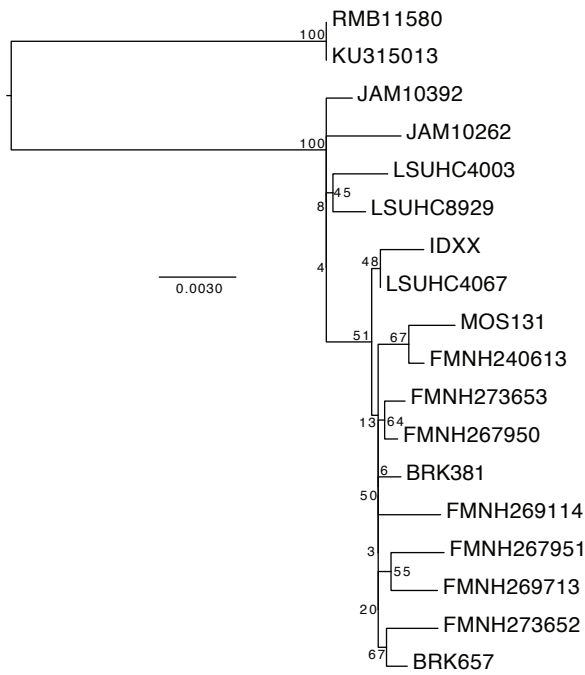

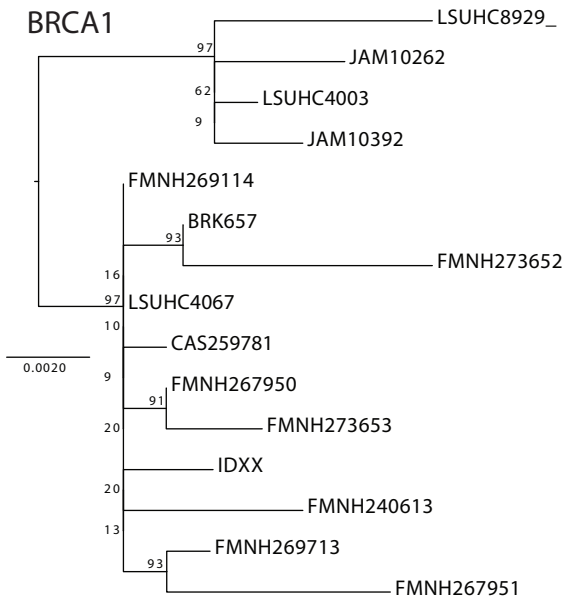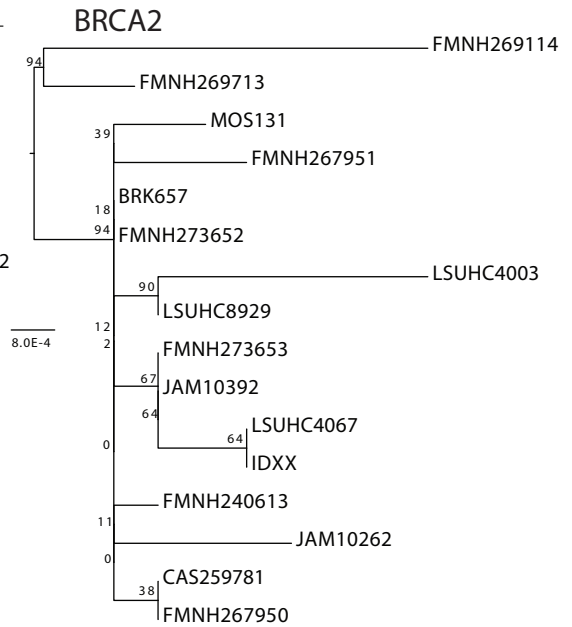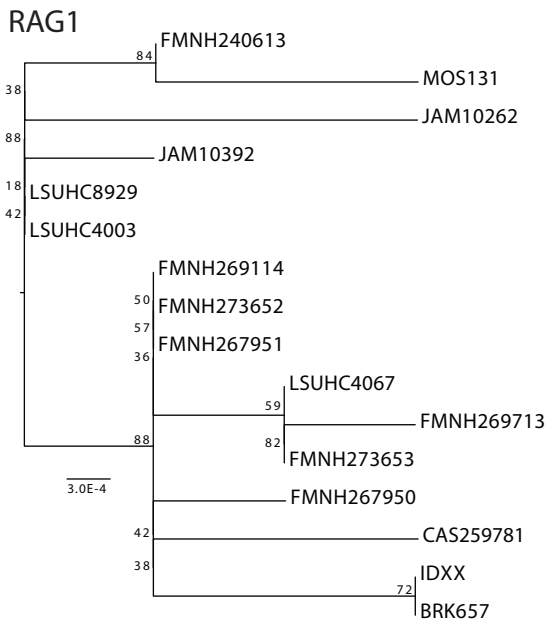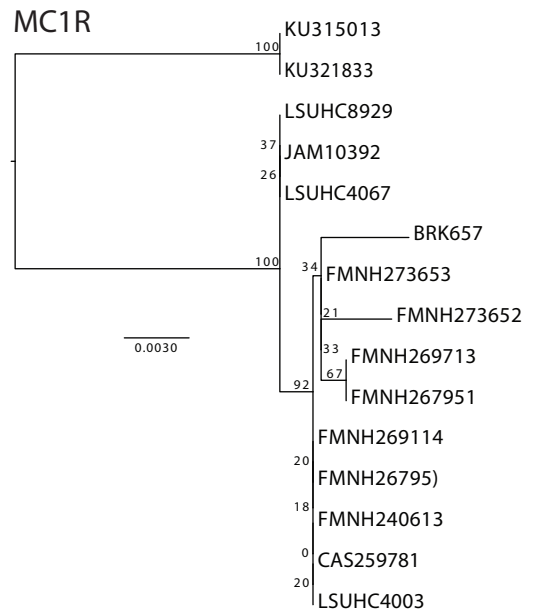

Supplement: Figure S2 [file peerj-05-3762-s002.pdf]
